# Psychometric properties of the German-language questionnaire for urinary incontinence diagnosis (QUID) in women with urinary incontinence

**DOI:** 10.1007/s00404-021-06167-8

**Published:** 2021-08-06

**Authors:** Florian Brandt, Erich-Franz Solomayer, Panagiotis Sklavounos

**Affiliations:** 1 Clinic for Gynecology, Obstetrics and Reproductive Medicine, Saarland University Hospital and Medical Faculty of Saarland University, Homburg, Germany; 2 Saarland University, Saarbrücken, Germany

**Keywords:** Diagnostic accuracy, Patient reported outcome measures, Stress urinary incontinence, Sensitivity and specificity, Urge urinary incontinence, Validation study

## Abstract

**Purpose:**

The aim of this study was to translate the questionnaire for urinary incontinence diagnosis (QUID) into German and to assess its psychometric properties in German-speaking women with urinary incontinence (UI). The QUID contains two subscales to measure symptom severity of stress urinary incontinence (SUI) and urge urinary incontinence (UUI) and to distinguish between both forms.

**Methods:**

A total of 161 women with UI completed the QUID and the King’s Health Questionnaire (KHQ), each in the German version. To examine construct validity Spearman’s correlation coefficients between both questionnaires were computed. Furthermore, the internal consistency (Cronbach’s alpha) of the QUID and its criterion validity were examined. Looking at criterion validity, sensitivity, specificity, ROC curves, and Youden-indexes were computed for both subscales.

**Results:**

The QUID showed good construct validity by strong correlations with related domains of the KHQ. Cronbach’s alpha values were good for both subscales of the QUID (SUI-subscale: 0.76; UUI-subscale: 0.86). Sensitivity and specificity were 83% (95% CI, 0.72–0.9) and 45% (95% CI, 0.25–0.67) for the SUI-subscale and 83% (95% CI, 0.7–0.91) and 56% (95% CI, 0.4–0.72) for the UUI-subscale. Youden-index was 0.28 for the SUI-subscale and 0.39 for the UUI-subscale at the given cut-off values.

**Conclusion:**

Psychometric properties of the German-language QUID are principally good and support its use in the German-speaking area. However, the modest specificity when distinguishing between SUI and UUI should be taken into account.

**Trial registration number:**

DRKS00018777 (date of registration: 16-January-2020).

## Introduction

Urinary incontinence (UI) is a ubiquitous issue in gynaecologic practice and puts a lot of distress on patients who are affected [1]. The most common forms are stress urinary incontinence (SUI), urge urinary incontinence (UUI), or related mixed forms (MUI) [2]. Since SUI and UUI may require a different treatment, it is essential to distinguish between both forms [1–3]. A simple and feasible method for this purpose is the Questionnaire for Urinary Incontinence Diagnosis (QUID). It is a patient-reported outcome measure (PROM) which consists of two subscales to identify SUI and/ore UUI if existing [4]. Each subscale consists of three items to measure the symptom severity of the respective type of UI. Values of each item range from zero ‘none of the time’ to five ‘all of the time’ [4]. Adding the values gives a score (0–15) for each subscale [4]. Values equal to or greater than four in the SUI-subscale indicate SUI, while values equal to or greater than six in the UUI-subscale indicate UUI [4, 5]. The original English version is recommended for use as basic diagnostic tool to distinguish between SUI, UUI, and MUI in urogynecologic practice as well as to measure patient reported outcomes within respective clinical trials [4, 5]. Furthermore, it enables women to assess their UI type on their own [6].

However, since every language area has its distinctions, it is necessary to re-validate patient questionnaires if translated what means to accurately check their psychometric properties. In this regard the following quality criteria regularly are of interest: reliability, validity, and, in case of patient questionnaires like the QUID, which are supposed to discriminate between certain conditions, diagnostic accuracy [7, 8]. Reliability describes the replicability of the results of a measuring instrument. It indicates the extent to which several measurements that were carried out under the same conditions agree with each other. Reliability can be estimated using various methods. With regard to patient questionnaires internal consistency regularly is examined. Internal consistency indicates the extent to which several items that are supposed to measure the same dimension within a multi-item scale are related to each other. Validity describes the accuracy to which the condition that is supposed to be measured actually is measured. Forms of validity that play a special role within psychometric assessment of questionnaires are construct and criterion validity. Construct validity is usually verified by comparing the questionnaire to a similar questionnaire which has already been proven, while criterion validity examines the extent to which the questionnaire’s results agree with external criteria like physician’s diagnosis. Diagnostic accuracy relates to the ability of the questionnaire to discriminate between certain conditions (e.g. two forms of UI).

While the QUID had been part of respective studies performed in women speaking Spanish [9], Chinese [10], Thai [11], or Persian [12], its applicability in German-speaking women has not yet been checked. Therefore, the aim of this study was to translate the QUID into German, evaluate its psychometric properties in women with UI, and support its use in the German-speaking area based on scientific evidence.

## Materials and methods

### Data collection

An online survey was mailed to 3500 women in Germany via a German health insurance company in August 2020. Contacted women were randomly selected out of women ≥ 18 years of age. Within the mailing all women were informed about the study’s aims and about voluntariness and anonymity of participation. Of course it was possible to forward the survey link to other people of the personal environment. All participants had to fully complete the survey, had to be female, had to suffer from UI, and had to be ≥ 18 years old to be included in this study (inclusion criteria). To prevent a high dropout rate, which is higher within online surveys anyway, the survey was done without a comprehensive inquiry of characteristics going beyond gender, few sociodemographics, and type of incontinence. Consequently there were no specific exclusion criteria. However, this was acceptable since no treatment was investigated in the course of this study and, therefore, no confounding variables in terms of any treatment effect had to be controlled. Informed consent was assumed by taking part and submitting the survey after completion.

### Measurements

Initially, some general information concerning age, size, weight, gender, existence, and duration of UI was requested. Diagnosis data were retrieved by asking every participant if the complaint has already been diagnosed by a physician and if ‘yes’, what kind of UI (SUI, UUI, MUI) was diagnosed if sufficiently well-known. UI diagnosis regularly includes a comprehensive anamnesis of symptoms, micturition, and medical history as well as a physical examination to assess urethral support, descensus of pelvic organs, and stress test. If UI is not adequately clarified afterwards, patients undergo additional testing, including voiding diaries, pad tests, urinalysis, ultrasound, or urodynamic tests. Retrieving diagnosis data made it possible to examine criterion validity of the QUID by comparing its results with the reported physicians’ diagnoses as referenceable ‘gold standard’ [4, 6]. In the main part of the survey the QUID and the King’s Health Questionnaire (KHQ) were filled in. The QUID was described in the introduction. It was translated into German based on the *ISPOR principles of good practice in the cross-cultural adaption process for patient-reported outcome measures* [13] passing through the following steps:

1. Forward translation: Initial translation of the QUID from English to German was performed in a team of German native speakers. All members were fluent in English. There were no substantial differences between the translations of the team members. Therefore it was no issue to obtain a single concerted version.
2. Back translation: The German version was submitted to another team of English native speakers for back translation. All members were fluent in German and had no prior knowledge of the measure. Again there were no substantial differences both between the translations of the team members and between their translations and the original version. Therefore the concerted version of step 1 seemed to be applicable.
3. Testing and revision: Finally, the concerted version and alternative wording was discussed with medical practitioners. The obtained version was handed out to a small group in order to check understandability and interpretation. No problems occurred and the German-language QUID was finalized.

Permission to use the QUID within this study was granted by its developers. The translated German-language version of the QUID can be found in the “Appendix”.

The KHQ was used as a reference tool to check the construct validity of the QUID. The KHQ is a disease-specific quality of life (QoL) measure within the scope of UI [14]. It has already been validated for many languages [15, 16] including German [17, 18]. Moreover, it is recommended for use both in practice and research by the *International Consultation on Incontinence (ICI)* with the highest recommendation rate [19]. It contains seven multi-item domains: Role Limitations, Physical Limitations, Social Limitations, Personal Relationship, Emotional Problems, Sleep and Energy Disturbances, Severity Measures and two single-item domains: General health Perception and Incontinence Impact [14, 20]. Additionally, the current version includes a symptom severity scale with ten items assessing the presence and relative severity of incontinence symptoms, including one item to measure ‘urine leakage at physical activity’ [20]. Concerning the German version the first four items within the symptom severity scale capture symptoms of overactive bladder (OAB) [17, 18]. The possible score for each subscale ranges from zero (best health perception) to 100 (worst health perception) [14, 20]. Deviating from this, the individual results of the ten items within the Symptom Severity Scale are just added and can reach a total value of 0–30 (possible values are 0–3 at each Item) [20].

### Statistical analysis

First, descriptive statistics (mean, standard deviation (SD)) were computed for sample characteristics. The values were grouped according to type of UI. Group differences were computed using Kruskal–Wallis test, a non-parametric alternative to one-way ANOVA [21]. Both questionnaires were then checked for normal distribution of their scores and descriptive statistics were computed for both questionnaires too. This allowed for checking the QUID for floor and ceiling effects (percentages of subjects with the lowest and the highest possible scores). To receive a first impression of the QUID’s diagnostic accuracy, group differences regarding its scores were examined as follows: women with SUI vs. women without SUI *and* women with UUI vs. women without UUI. Hereto non-parametric Wilcoxon–Mann–Whitney test [22] was used.

Internal consistency was determined using Cronbach’s alpha. Cronbach’s alpha measures the extent to which the items of a subscale are related to one another (interrelatedness) [23]. It was computed for the QUID’s subscales and the entire QUID. A value of ≥ 0.7 was considered acceptable [7]. Additionally, Spearman correlation coefficients between the responses of each individual item and the corresponding total score of the associated subscale ommitting that item were computed.

Construct validity is confirmed if there is strong correlation with a test which measures the same construct (convergent validity) and weak correlation with a test which measures another construct (discriminant validity). To test construct validity of the QUID its correlation with the KHQ was examined. Since its scores had non-normal distribution, Spearman’s rho (*r*_s_), a non-parametric correlation test, was used to compare the responses. Spearman’s rho was computed both for the overall QUID and its subscales with each domain of the KHQ. Furthermore, correlations between the subscales of the QUID were computed. Values from 0.1 to 0.3 were considered weak, 0.3 to 0.5 moderate, and ≥ 0.5 strong [24].

To examine criterion validity and diagnostic accuracy, sensitivity (SE) and specificity (SP), including 95% confidence intervals, were calculated for each subscale using the reported physicians’ diagnoses as reference value. In addition, we drew a ROC-curve (receiver operating characteristic curve) for each subscale. A ROC-curve is a graph showing the performance of a classification model at all classification thresholds meaning all possible cut-off scores. It plots the true positive rate (= sensitivity) at the ordinate and the false positive rate (= 1 − specificity) at the abscissa (*x*-axis) of a two-dimensional graph [25]. In addition to this Youden’s J (also called Youden’s index) was calculated. Youden’s J is a measure to determine which cut-off score is most qualified to distinguish between the two groups (non-SUI vs. SUI, respectively, non-UUI vs. UUI). It is calculated as follows: Youden’s J = sensitivity + specificity – 1. The higher Youden’s J, the higher the performance of the related cut-off score in terms of the combination of sensitivity and specificity [26]. Finally, the hit ratio for the overall QUID at the given cut-off scores was computed. At computation of the hit ratio a match with physician diagnosis in both subscales was counted as ‘correct’ (= 1), a match in just one subscale as ‘partly correct’ (= 0.5), and no match as ‘wrong’ (= 0).

The statistical analysis was performed using *R*, a free software environment for statistical computing and graphics.

### Sample size calculation

To examine the interesting group differences between women with and without UUI, which were expected to be large [5], with a power (1 − β) of 0.8 and α = 0.05, a minimum sample size of 32 women with UUI and 24 women without UUI was required (computed with G*Power [27]; test: Wilcoxon–Mann–Whitney test (two groups); tails: two; effect size: 0.8 [24]; allocation ratio: 53/39 (based on current allocation)). To assess the interesting group differences between women with and without SUI, which were expected to be large too, with a power of 0.8 and α = 0.05, a minimum sample size of 56 women with SUI and 18 women without SUI was required (computed with G*Power [27]; test: Wilcoxon–Mann–Whitney test (two groups); tails: two; effect size: 0.8 [24]; allocation ratio: 70/22 (based on current allocation)).

To compute the interesting correlation coefficients between comparable measures, which were expected to be strong [5], with a power of 0.8 and α = 0.05, a minimum sample size of 26 was required (computed with G*Power [27]; test: linear bivariate regression, one group; tails: two; correlation: 0.5 [24]; SD (*x*): 30 [18]; SD (*y*): 3 [5]).

## Results

### Study population

Of the 3500 contacted women 246 (7%) took part in the anonymous online survey carried out in August/September 2020. Thereof 161 (65%) met the inclusion criteria and were included in this study. Meaning all of the included women fully completed the survey, were ≥ 18 years old, and had UI, of which 92 (57%) reported the type of UI diagnosed by a physician: 39 SUI (42%), 22 UUI (24%), and 31 MUI (34%). In 69 participants the type of UI was not recorded because it wasn’t sufficiently well known. Since respective data are necessary to consider sensitivity and specificity, only 92 of 161 women were included in this part. An overview of the sample characteristics can be found in Table 1. There were no significant differences between groups concerning age, weight, height, or BMI.

**Table 1:** Sample characteristics (mean ± SD or *n* (%))

	SUI patients (*n* = 39)	UUI patients (*n* = 22)	MUI patients (*n* = 31)	*p*-value (*H*-test)	Overall (*n* = 161)
Age (years)	54.2 ± 10.4	53.1 ± 12.2	58.1 ± 9.4	0.229	56.2 ± 10.2
Height (cm)	167.7 ± 6.5	166.7 ± 7.1	166.8 ± 6.7	0.494	167.6 ± 6.5
Weight (kg)	82.7 ± 17.9	85.1 ± 22.4	85.9 ± 21.9	0.872	85.5 ± 21.4
BMI	29.5 ± 6.7	30.6 ± 8.0	30.9 ± 8.0	0.831	30.5 ± 7.6
Duration of UI					
< 1 year	4 (10%)	4 (18%)	2 (6%)	–	19 (12%)
1–3 years	9 (23%)	11 (50%)	12 (39%)	–	65 (40%)
> 3 years	26 (67%)	7 (32%)	17 (55%)	–	77 (48%)

*BMI* body mass index; *cm* centimeter; *kg* kilogram; *MUI* mixed urinary incontinence; *n* sample size; *SD* standard deviation; *SUI* stress urinary incontinence; *UI* urinary incontinence; *UUI* urge urinary incontinence

### Outcomes and internal correlations

Shapiro–Wilk test of normality [28] showed that neither the scores of the QUID, nor of the KHQ were normally distributed (*p* < 0.05 in each case).

Table 2 shows the mean scores of the QUID and the KHQ according to type of UI and overall. Women with SUI had a significantly higher score in the SUI-subscale of the QUID (α ≤ 0.05) while women with UUI had a significantly higher UUI-score (α ≤ 0.01). The same applies to related domains of the KHQ. Overall, the mean scores were higher in women with UUI or MUI than in women with SUI. Only in the KHQ item ‘Urine leakage at physical activity’ as well as in the SUI-Subscale of the QUID mean scores were higher in women with SUI.

**Table 2:** KHQ and QUID scores (mean ± SD)

	SUI patients (*n* = 39)	UUI patients (*n* = 22)	MUI patients (*n* = 31)	*p*-value with vs. without SUI	*p*-value with vs. without UUI	Overall (*n* = 161)
KHQ						
General health perception	34.6 ± 27.3	47.7 ± 24.3	46.0 ± 25.9	0.213	0.037	42.2 ± 25.5
Incontinence impact	71.8 ± 31.1	83.3 ± 22.4	88.2 ± 25.2	0.816	0.023	71.9 ± 30.0
Role limitations	54.2 ± 30.8	75.0 ± 26.6	77.9 ± 26.3	0.184	< 0.01	60.9 ± 31.1
Physical limitations	48.7 ± 26.9	58.3 ± 35.6	72.0 ± 28.7	0.974	< 0.01	51.6 ± 31.3
Social limitations	28.2 ± 31.5	32.5 ± 33.6	46.7 ± 32.2	0.668	0.071	30.2 ± 31.8
Personal relationship^a^	35.1 ± 25.4	51.3 ± 25.0	41.6 ± 32.4	0.125	0.134	36.4 ± 28.2
Emotional problems	44.4 ± 34.4	60.1 ± 31.7	60.5 ± 31.3	0.331	0.030	44.4 ± 33.1
Sleep and energy disturbances	30.7 ± 29.5	56.0 ± 33.6	57.5 ± 33.9	0.083	< 0.01	39.5 ± 31.6
Severity measures	69.7 ± 23.3	72.7 ± 18.4	78.7 ± 18.8	0.629	0.227	70.5 ± 21.9
Overactive bladder^b^	42.8 ± 29.8	80.7 ± 24.6	79.8 ± 23.5	< 0.01	< 0.01	58.4 ± 32.0
Symptom severity scale	11.6 ± 6.1	15.1 ± 5.5	15.3 ± 5.6	0.176	< 0.01	12.9 ± 6.2
Urine leakage at physical activity	2.3 ± 0.8	1.5 ± 1.2	2.1 ± 1.0	< 0.01	0.049	2.1 ± 1.0
QUID						
SUI-Subscale	7.5 ± 3.1	5.1 ± 4.5	7.2 ± 3.8	0.014	0.099	6.4 ± 3.8
UUI-Subscale	5.9 ± 3.7	8.4 ± 3.2	9.3 ± 3.7	0.249	< 0.01	7.0 ± 3.8
Overall	13.4 ± 5.2	13.5 ± 5.8	16.4 ± 5.8	0.244	0.211	13.4 ± 5.9

*KHQ* King’s Health Questionnaire; *n* sample size; *QUID* Questionnaire for Urinary Incontinence Diagnosis; *SD* standard deviation; *SUI* stress urinary incontinence; *UUI* urge urinary incontinence

^a^Because of missing values *n* = 109 in this domain (SUI patients: *n* = 28; UUI patients: *n* = 13; MUI patients: *n* = 22)

^b^Because of missing values *n* = 158 in this domain (SUI patients: *n* = 38; UUI patients: *n* = 22; MUI patients: *n* = 31)

A maximum of *n* = 7 (4%) of participants’ scores clustered to the bottom end (floor) and a maximum of *n* = 3 (2%) to the top end (ceiling) of the QUID. The maximum at the bottom end occurred in the SUI-subscale while the maximum at the top end occurred in the UUI-subscale.

The internal correlations of the QUID are shown in Table 3. In addition to respective values in Table 3, Cronbach’s alpha was 0.75 (95% CI, 0.69–0.81) for the overall QUID.

**Table 3:** Internal correlations of the QUID

	Corr. with SUI-scale^a^	Corr. with UUI-scale^b^	Cronbach’s alpha
Item 1	0.57^d^	0.04	–
Item 2	0.63^d^	0.36^d^	–
Item 3	0.55^d^	0.11	–
SUI-scale	1.0^d^	0.18^c^	0.76 (0.7–0.83)^e^
Item 4	0.21^d^	0.7^d^	–
Item 5	0.15	0.75^d^	–
Item 6	0.13	0.74^d^	–
UUI-scale	0.18^c^	1.0^d^	0.86 (0.82–0.89)^e^

*Corr*. correlation; *n* sample size; *P P*-value; *QUID* Questionnaire for Urinary Incontinence Diagnosis; *SUI* stress urinary incontinence; *UUI* urge urinary incontinence

^a^Item 1–3: Spearman correlation between individual item response and the SUI-score omitting that item. Rest: Overall Spearman correlation

^b^Item 4–6: Spearman correlation between individual item response and the UUI-score omitting that item. Rest: Overall Spearman correlation

^c^*p* < 0.05

^d^*p* < 0.01

^e^95% confidence interval in brackets

### Correlations between QUID and KHQ

Spearman’s correlation coefficients and related *p*-values are shown in Table 4. The SUI-Subscale of the QUID was strongly correlated to the single item ‘urine leakage at physical activity’ (*p* < 0.01) and moderately correlated with the domains ‘Incontinence impact’, ‘Role limitations’, ‘Physical limitations’, ‘Severity measures’, and the ‘Symptom severity scale’ (*p* < 0.01). There were low correlations (*p* < 0.01) with ‘Social limitations’, ‘Personal relationship’, and ‘Emotional problems’ and no correlations with ‘General health perception’, ‘Sleep and energy disturbances’, and ‘Overactive bladder’. The UUI-Subscale of the QUID significantly was correlated with every domain of the KHQ (*p* < 0.01) except ‘urine leakage at physical activity’. There were low correlations with ‘General health perception’, moderate correlations with ‘Social limitations’, ‘Personal relationship’, and ‘Emotional problems’ and high correlations with the remaining domains. The overall QUID also correlated with every domain of the KHQ at a significance level of *α* ≤ 0.01 respectively *α* ≤ 0.05 (only for the domain ‘General health perception’). There was a low correlation with ‘General health perception’ and moderate to high correlations with the remaining domains.

**Table 4:** Correlations between QUID and KHQ (Spearman’s rho)

	QUID SUI score	QUID UUI score	QUID total score
KHQ			
General health perception	0.02	0.26^b^	0.19^a^
Incontinence impact	0.38^b^	0.54^b^	0.61^b^
Role limitations	0.38^b^	0.57^b^	0.61^b^
Physical limitations	0.4^b^	> 0.5^b^	0.59^b^
Social limitations	0.24^b^	0.47^b^	0.45^b^
Personal relationship	0.27^b^	0.31^b^	0.4^b^
Emotional problems	0.27^b^	0.4^b^	0.45^b^
Sleep and energy disturbances	0.09	0.53^b^	0.41^b^
Severity measures	0.41^b^	0.45^b^	0.55^b^
Overactive bladder	0.02	0.6^b^	0.38^b^
Symptom severity scale	> 0.3^b^	0.54^b^	0.54^b^
Urine leakage at physical activity	0.65^b^	0.02	0.43^b^

*KHQ* King’s Health Questionnaire; *n* sample size; *p p*-value; *QUID* Questionnaire for Urinary Incontinence Diagnosis; *SUI* stress urinary incontinence; *UUI* urge urinary incontinence

^a^*p* < 0.05

^b^*p* < 0.01

### Sensitivity, specificity, and ROC curves

**Fig. 1:**
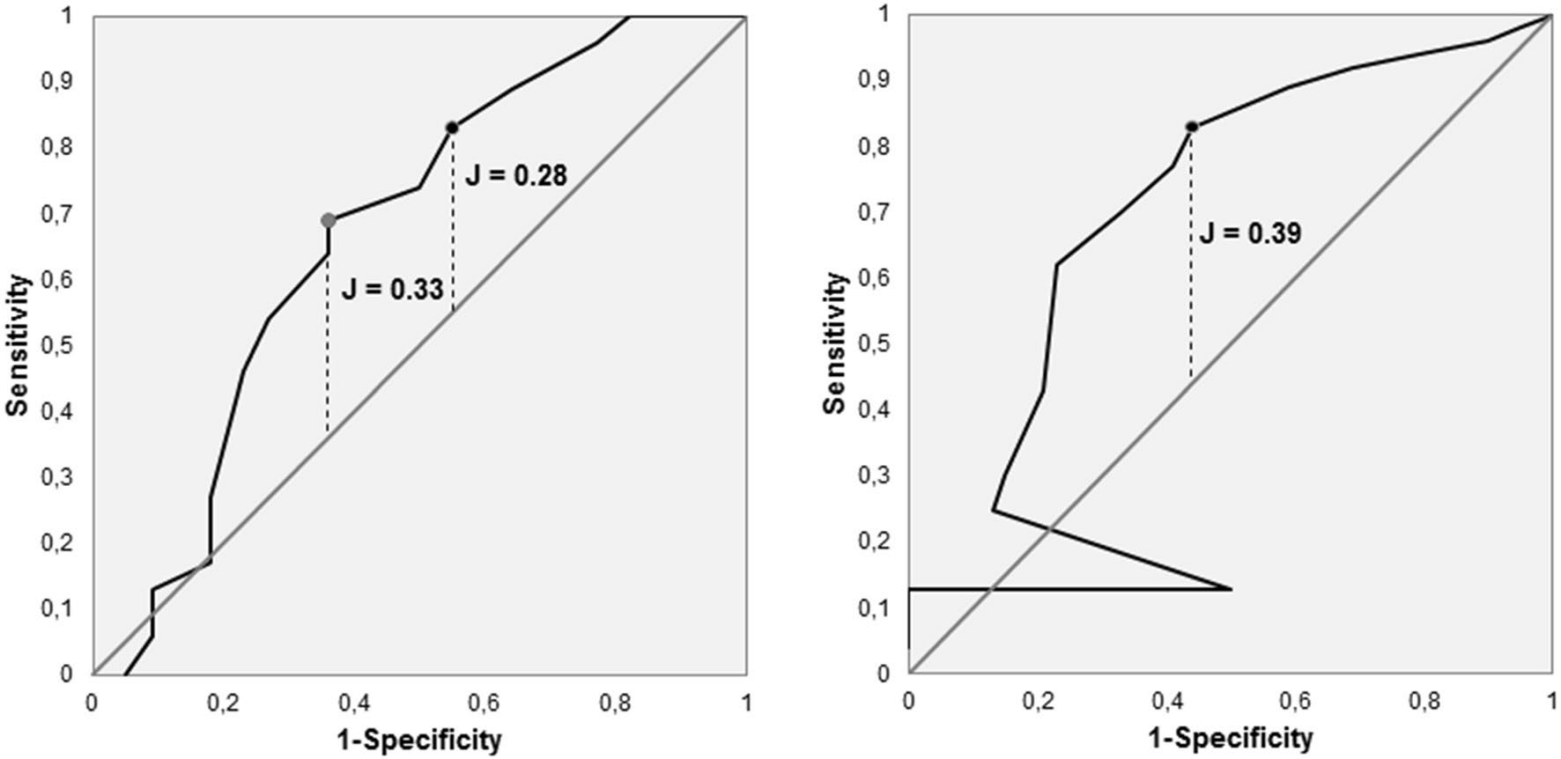
ROC curves for the SUI-subscale (left) and the UUI-subscale (right). The ROC curves are delineated in black. The black points on the ROC curves represent the sensitivity/1-specificity-combinations for the given cut-off values. Related Youden’s J is given too. Since the given cut-off value of the SUI-subscale (≥ 4) didn’t result in an optimum of Youden’s J, the left grey point shows the sensitivity/1-specificity-combination where Youden’s J reaches an optimum (cut-off value ≥ 6). The diagonal represents decisions based purely on chance

Sensitivity (SE) and Specificity (SP) were calculated both for the SUI- and the UUI-subscale of the QUID. Regarding the SUI-Subscale (given cut-off score ≥ 4) the following results were obtained: SE = 0.83 (95% CI, 0.72–0.9) and SP = 0.45 (95% CI, 0.25–0.67). Corresponding results for the UUI-Subscale (given cut-off score ≥ 6) were: SE = 0.83 (95% CI, 0.7–0.91) and SP = 0.56 (95% CI, 0.4–0.72). The ROC-curves are shown in Fig. 1. The given classification threshold of the SUI-Subscale did not result in an optimum regarding Youden’s J (0.28 for the given cut-off score ≥ 4). A cut-off score ≥ 6 would result in Youden’s J = 0.33, SE = 0.69 (95% CI, 0.56–0.79), and SP = 0.64 (95% CI, 0.41–0.82). The given cut-off score ≥ 6 in the UUI-Subscale resulted in an optimum of Youden’s J (0.39).

Hit ratio for the overall QUID was 73% at the given cut-off scores.

## Discussion

The aim of this study was to assess the psychometric properties of the German-language QUID in women with UI. Required data were collected in 161 appropriate women which made it possible to examine internal consistency, construct validity, and criterion validity of the QUID. The proportion of UI types in diagnosed women in this sample closely corresponds to the proportion measured within large-scaled prevalence studies [29, 30] with a slight overemphasis of UUI. This speaks of the representativity of the following findings.

### Internal consistency

The scores’ distributions gave no indication of floor or ceiling effects. Both subscales of the QUID showed good internal consistency with Cronbach’s alpha coefficients above the accepted standard of 0.7. This finding is confirmed by the strong correlations between the individual items of the QUID and their related subscale (item-total-correlations). The internal consistency is similar to that of the original English version: Cronbach’s alpha is the same for the overall QUID (0.75), a little lower in the UUI-subscale (0.86 vs. 0.87), but clearly higher in the SUI-subscale (0.76 vs. 0.64) [5]. Cronbach’s alpha was even higher in the Chinese (SUI: 0.91; UUI: 0.89) [10] and Spanish version (both subscales: 0.94) [9]. Item-total-correlations concerning the UUI-subscale were similar to those of the original English version [5]. Item-total-correlations concerning the SUI-subscale were higher than those in the original version with all correlation coefficients > 0.5, which is considered as strong [24]. Altogether, reliability of the German-language QUID is good.

### Construct validity

There was a weak correlation with the KHQ domain ‘General health perception’. Between the SUI-subscale and ‘General health perception’ there even was no correlation. Since general health measures are not closely related to disease-specific measures [17], this discrepancy speaks of the discriminant validity of the QUID. Furthermore, there was no correlation between the SUI-subscale and the KHQ domain ‘Sleep and energy disturbances’. This might be due to the fact that symptoms that disturb sleep, such as nocturia, are more likely to be found in UUI [2]. Because an overactive bladder syndrome is necessary for diagnosis of UUI [2], it is coherent that there was no correlation between the KHQ’s OAB-domain and the SUI-subscale. The same applies to the non-existing correlation between the UUI-subscale and ‘urine leakage at physical activity’, because urine leakage at physical activity is a generic symptom of SUI [1–3]. The weak correlation between the QUID’s subscales speaks of its ability to discriminate between SUI and MUI.

Evidence of convergent validity is provided by strong correlation of the QUID’s UUI-subscale with the KHQ’s OAB-domain as well as by the strong correlation of the QUID’s SUI-subscale with the KHQ’s item ‘urine leakage at physical activity’, which is characteristic of SUI [1–3]. QUID-KHQ-correlation has never been tested before. Hence a straight comparison with findings of further studies is not possible. However, score-correlations of the English-language QUID with corresponding items of the Urinary Distress Inventory (UDI), another commonly used UI-specific QoL-measure [31], were similar (both 0.68) [5].

### Criterion validity and diagnostic accuracy

A first indication of the QUID’s ability to discriminate between SUI and UUI is given by the mean scores of the UUI-subscale, which were significantly higher in women with UUI than in women without UUI. The same applies to the mean scores of the SUI-subscale and women with vs. without SUI. Concerning sensitivity both subscales were similar to the English-language version (0.83 vs. 0.85 for SUI and 0.83 vs. 0.79 for UUI) [4]. However, specificity (0.45 vs. 0.71 for SUI and 0.56 vs. 0.79 for UUI) and total hit ratio (73% vs. 80%) were better in the English-language version [4]. This might be due to differences in the spectrum of disease between the studied populations which can result in a variation of sensitivity and/or specificity [8].

The ROC curves show that the given cut-off score (≥ 6) leaded to an optimum in the UUI-subscale. In the SUI-subscale the given cut-off score (≥ 4) did not lead to an optimum. Here the ROC curve has an optimum at a cut-off score of ≥ 6. A cut-off score of ≥ 6 in the SUI-subscale would enhance specificity to 0.64 but simultaneously would reduce sensitivity to 0.69. Considering urogynecologic practice such an adjustment of the cut-off score cannot be recommended without reserve. Since all patients often benefit from initial treatment that is focused on UUI [2], some false-positives in the UUI-subscale inherently are acceptable. The same applies to the SUI-subscale, because pelvic floor muscle training (PFMT), which is very-low risk, is the first choice therapy for SUI [1–3]. Therefore, it could be more important to gain sensitivity than specificity. The poorer performance of the ROC curve on the SUI-subscale could be due to the fact that the participants with SUI, according to the mean scores of the KHQ, had lower degrees of severity.

### Clinical implications and limitations

The German-language QUID enables quick and simple assessment of SUI- and/or UUI-symptoms in German-speaking women. At this it is much more specific than pre-existing German-language UI-questionnaires like the KHQ which has a broader and more general perspective on UI-related symptoms and QoL [14]. Therefore, the QUID currently is the most qualified PROM which was psychometrically evaluated in the German-speaking area to assess SUI- and/or UUI-symptoms. When used within respective clinical trials it focuses on SUI- and/or UUI-specific outcomes, while blinding out other dimensions [4]. Thus it brings out measurement results which are as precise as possible [32] and simultaneously are easy to interpret. The German-language QUID is also useful within urogynecologic practice as it gives a first impression of SUI- and/or UUI-symptoms in a standardized manner for the doctor but also for the patient. In particular it is suitable to assess symptom severity. It also can be used as a supportive tool to discriminate between SUI and UUI but positive results should be considered with caution, since specificity was not sufficient in both subscales.

Although this study created comprehensive knowledge about the psychometric properties of the German-language QUID, there were certain limitations. Since the online survey was performed anonymously, test–retest reliability could not be measured. Furthermore, sensitivity to change, which is the ability of an instrument to measure a change in condition, of the QUID was not assessed. This is due to the current coronavirus pandemic. To assess sensitivity of change study participants regularly obtain an intervention which is known as effective. Within the scope of UI this includes PFMT, bladder training (both professionally accompanied by a physical therapist), or surgical interventions (especially mid-urethral sling placement to treat SUI) [2]. Physiotherapy practices have been closed during coronavirus pandemic and many surgical interventions have been delayed to hold capacities for COVID-19-patients. However, sensitivity to change is indicated by strong correlations with related domains of the German-language KHQ, which already has shown its suitability both as research assessment and therapeutic monitoring tool by good sensitivity to change [17, 18]. Nevertheless, further research could provide evidence of the German-language QUIDs’ sensitivity to change by applying it as outcome measure within an interventional study in the context of UI.

## Conclusions

This evaluation of the German-language QUID in a sample of 161 women with UI indicated that its psychometric properties are good and, therefore, support its use both as basic diagnostic tool to distinguish between SUI, UUI, and MUI, and as patient-reported outcome measure in German-speaking populations. An examination of construct validity presented moderate to strong correlations with related domains of the KHQ. Both subscales of the German-language QUID had relatively high internal consistency. Additionally, it is able to provide an initial assessment of the type of UI. When distinguishing between SUI and UUI, however, the modest specificity should be taken into account.

## Figures and Tables

**Table 5:**
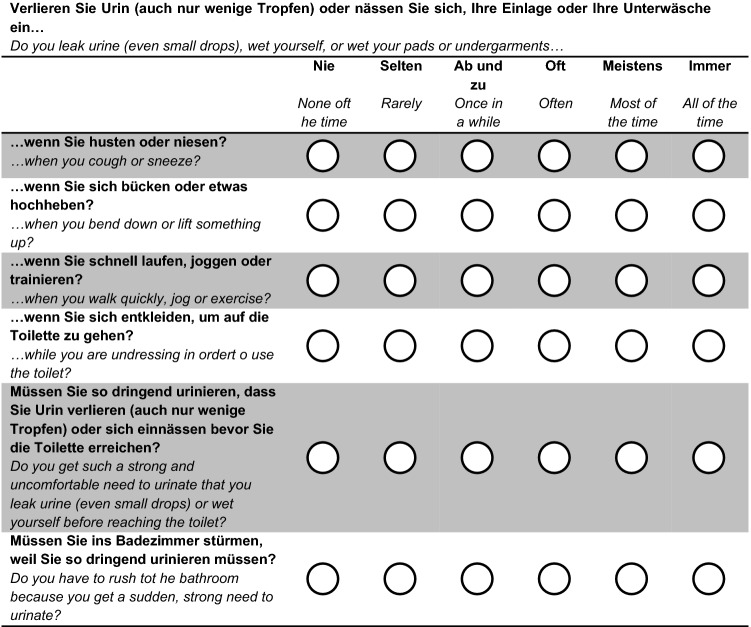
The German-language Questionnaire for Urinary Incontinence Diagnosis (QUID—German version)

Scoring: Each item scores 0 (None of the time), 1 (Rarely), 2 (Once in a while), 3 (Often), 4 (Most of the time) or 5 (All of the time). Responses for items 1, 2, and 3 are summed for the SUI score. Responses for items 4, 5, and 6 are summed for the UUI score. Both scores have to be summed for the overall score

## Data Availability

Not applicable.

## Appendix

See Table 5.
